# Group‐based interventions to reduce diabetes distress in adults with type 1 diabetes: A rapid realist review of the Reduce and TunedIn interventions

**DOI:** 10.1111/dme.70242

**Published:** 2026-02-12

**Authors:** Sarah Sims, Vibeke Stenov, Mette Due‐Christensen, Lawrence Fisher, Clara Fabian‐Therond, Megan Peck, Shalini Ahuja, Siobhan O'Connor, Ruth Abrams, Jennifer Halliday, Richard I. G. Holt, Jane Speight, Jackie Sturt, Ruth Harris, D‑Stress Study Group, Iliatha Papachristou Nadal, Jennifer Mohammadi, Jorg Huber, Gary Hickey, Pratik Choudhary, Sufyan Hussain, Kate Hardenberg, Francesca Fiorentino, Samuel Watson, Efrat Lusky, Rosie Walker, Ramzi Ajjan, James Shearer Marietta Stadler

**Affiliations:** ^1^ King's College London London UK; ^2^ NAMU ‐ National Center for Applied Research in Older Citizens Living with Multimorbidity Univeristy College Absalon Denmark; ^3^ Steno Diabetes Center Copenhagen, Region H Copenhagen Denmark; ^4^ University of California San Francisco California USA; ^5^ University of Surrey Guildford UK; ^6^ School of Psychology, Institute for Health Transformation Deakin University Geelong Victoria Australia; ^7^ The Australian Centre for Behavioural Research in Diabetes, Diabetes Victoria Carlton Victoria Australia; ^8^ University of Southampton Southampton UK

**Keywords:** diabetes distress, realist process evaluation, realist review, type 1 diabetes

## Abstract

**Aims:**

Diabetes distress is highly prevalent among adults with type 1 diabetes, yet remains under‐recognised and rarely addressed in routine diabetes care. This rapid realist review aimed to identify how, why, for whom, and in what contexts group‐based interventions reduce elevated diabetes distress in adults with type 1 diabetes, to inform the co‐adaption of a new diabetes distress intervention in the UK (‘D‐Stress Reduce’).

**Methods:**

Following established rapid realist review methods, we synthesised evidence from 27 papers relating to two existing diabetes distress interventions and the psychological and social theories underpinning them. Data extraction and analysis focused on identifying contexts, mechanisms and outcomes, supported by stakeholders including methodological experts and programme architects.

**Results:**

Seven programme theories were generated and articulated through 20 ‘If‐Then‐Because’ statements. Key theories highlighted emotional regulation, peer support and person‐centred facilitation in reducing diabetes distress. Other theories included regular assessment and follow‐up, motivation for action, and empowerment in self‐management. The review found that interventions were most effective when adults with type 1 diabetes reported feeling emotionally safe, listened to and respected, and when group facilitators shifted from directive, hierarchical communication styles to collaborative approaches. Contextual factors, such as group composition, facilitator skill, and individual readiness for change influenced outcomes.

**Conclusions:**

This review provides a theory‐driven foundation for developing, implementing and evaluating a new co‐adapted diabetes distress intervention in the UK (‘D‐Stress Reduce’) and highlights multiple components that can be addressed to improve the care pathway and group‐based experience.

**What's new?**

**What is already known?**

- Diabetes distress is common among adults with type 1 diabetes, impairs quality of life and self‐management behaviours, and is associated with suboptimal glycaemic levels.
- Diabetes distress remains under‐recognised and rarely addressed in routine diabetes care.

**What has this study found?**

- This review supports the design of ‘D‐Stress Reduce’, a co‐adapted intervention to manage elevated diabetes distress in the UK.
- Seven programme theories and 20 ‘If‐Then‐Because’ statements were developed to guide future intervention development and evaluation.

**What are the implications of the study?**

- This review provides a theory‐driven foundation for implementing and evaluating the D‐Stress Reduce intervention.

## 1 INTRODUCTION

Diabetes distress refers to the emotional burden associated with the daily management of diabetes. Meta‐analyses indicate that approximately one in three adults living with type 1 or type 2 diabetes experience elevated diabetes distress.^1^ Most studies have been conducted in high‐income countries, where the prevalence rates appear consistent. More recent evidence suggests that diabetes distress may be even more widespread, with up to 80% of adults with diabetes reporting at least one ‘problem area’ that could contribute to distress.^2^ Diabetes distress is linked to reduced quality of life, impaired self‐care behaviours and suboptimal glycaemic levels,^3^, ^4^, ^5^ yet, it remains under‐recognised and rarely addressed as part of routine diabetes care.^2^, ^6^, ^7^ Diabetes distress has become a greater focus for diabetes researchers and funders recently, with specific funding calls being launched by the USA National Institutes of Health and the UK National Institute for Health and Care Research and Diabetes UK. International clinical guidelines for type 1 diabetes and type 2 diabetes have advocated for yearly assessment of the emotional health of people with diabetes.^8^, ^9^ The European Association for the Study of Diabetes (EASD) has developed the world's first evidence‐based Clinical Guideline for the assessment and management of diabetes distress in adults with type 1 and type 2 diabetes, which is due to be published in early 2026 (www.easd.org/guidelines/statements‐guidelines/diabetes‐distress). One research study working to develop a UK care pathway for type 1 diabetes is the D‐Stress study (https://www.kcl.ac.uk/research/d‐stress‐study), which aims to develop and integrate a new, innovative care pathway to manage diabetes distress in the National Health Service (NHS). The D‐Stress study did not include adults with type 2 diabetes (who also experience diabetes distress), as contextual factors are likely to differ considerably.^10^

One component of the D‐Stress study (the ‘D‐Stress Reduce’ intervention) aims to address elevated diabetes distress through a group‐based psychological intervention. The intervention will be co‐adapted using the frameworks, content and structure from two existing group‐based interventions with an interventional focus on, and primary outcome of, diabetes distress reduction in type 1 diabetes—the United States (US) intervention, ‘TunedIn’, and the Danish intervention, ‘Reduce’. Although there has been a growing number of meta‐analyses on the effects of interventions aimed at reducing diabetes distress among adults living with type 1 diabetes or type 2 diabetes,^11^, ^12^, ^13^ intervention studies specifically designed to target diabetes distress in people with type 1 diabetes remain scarce. To the best of our knowledge, TunedIn and the Danish Reduce are among the few diabetes‐tailored group psychological interventions specifically aimed at reducing elevated diabetes distress as their primary outcome. Blood glucose reduction was a target for TunedIn but not for the Danish ‘Reduce’. Furthermore, these interventions were chosen because the D‐Stress study involves international investigators who have successfully led diabetes distress targeted research in type 1 diabetes using the same approaches but in different healthcare contexts.^14^, ^15^, ^16^ Our interest in understanding what works, for whom and in what contexts, means that these interventions (which are similar in underpinning theory and delivery but within very different contexts) made them ideal interventions on which to base our UK‐focused research in the NHS setting.

TunedIn is an emotion‐focused, psychologist‐led intervention aimed at reducing diabetes distress in adults with type 1 diabetes and elevated HbA_1c_. Underpinned by Acceptance and Commitment Therapy, TunedIn is a time‐limited, group‐based psychological intervention with no diabetes self‐management educational content, delivered online over 3 months.^15^ TunedIn was evaluated and found to yield significant reductions in both diabetes distress and HbA_1c_.^15^ However, TunedIn is primarily conducted by trained diabetes psychologists in controlled settings. The intervention was subsequently revised and refined as a psychoeducational intervention for adults with type 1 diabetes in a Danish publicly funded healthcare delivery context and was renamed ‘Reduce’. What Reduce adds is the integration of the perspectives of adults with type 1 diabetes, clinical health care professionals, and relevant experts within the field to further design, develop, and adjust the intervention into a pragmatic intervention designed to integrate diabetes distress support into publicly funded hospitals. Reduce is also underpinned by Acceptance and Commitment Therapy, in addition to other psychological and social theories, including Self‐Determination Theory, Social Comparison Theory, and Social Learning Theory. The Danish intervention is delivered in‐person via five bi‐weekly small‐group sessions facilitated by two trained diabetes nurse specialists.^17^ A pilot study found that participation in the Danish Reduce intervention was associated with a significant reduction in diabetes distress and a significant increase in quality of life.^16^ Whilst TunedIn and the Danish Reduce have similar psychological groundings and have both been found to significantly reduce diabetes distress, there are some notable differences between the two interventions. For example, Reduce is delivered in person (rather than online) at the diabetes clinic and includes dialogue tools and curriculum to inform and assist diabetes specialist nurses (rather than psychologists) in delivering the intervention. This is partially due to the substantial variety between the healthcare contexts in the US and Denmark in terms of structure, funding, governance and accessibility.

This article discusses the findings of a rapid realist review undertaken as part of the co‐adaption process for developing the D‐Stress Reduce intervention for the UK healthcare setting. A rapid realist review is an evidence synthesis approach that applies realist principles in a time‐sensitive manner to inform policy and practice. Rapid realist reviews are particularly suited for complex interventions where decision makers require timely insights without sacrificing explanatory depth. Unlike traditional realist reviews, which can be resource‐intensive and lengthy, a rapid realist review streamlines the process by engaging content experts and stakeholders to prioritise relevant evidence.^18^ The aim of this rapid realist review was to identify the mechanisms through which TunedIn and Danish Reduce were assumed to reduce elevated diabetes distress and the contexts thought to influence their effectiveness. The findings are being used to inform and support the development of the D‐Stress Reduce intervention in the UK, enabling researchers to generate hypotheses around who benefits and why. These hypotheses will be interrogated and tested in subsequent phases of the study as part of a realist process evaluation.

## 2 METHODS

**FIGURE 1:**
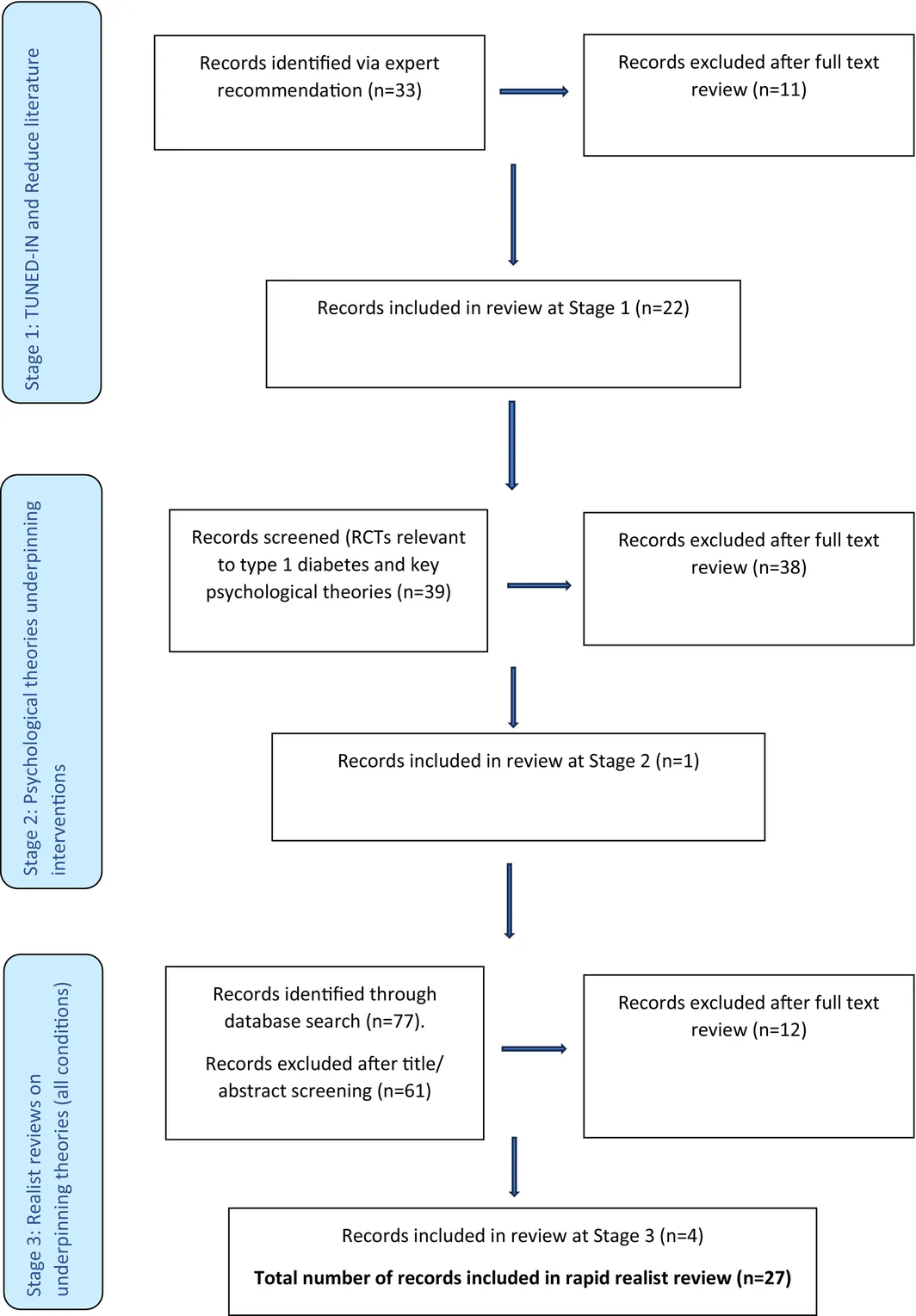
Flowchart of rapid realist review process.

A rapid realist review methodology was selected due to the time‐sensitive nature of the study. Evidence‐informed guidance for conducting rapid realist reviews was followed^18^ to ensure that quality and publication standards in realist reviewing were upheld.^19^, ^20^ Box 1 provides definitions of how the terms, ‘Programme Theory’, ‘Context’, ‘Mechanism’ and ‘Outcome’ were used in this review.^21^ A flow diagram for the review process is provided in Figure 1.

BOX 1 **Definitions of realist terms and how they were used in this rapid realist review**

**Programme theory:** The programme theory specifies what is supposed to be done in programme (theory of action) and how and why that is expected to work (theory of change).^21^

**Context (C):** *‘the “backdrop” of programmes and research… broadly understood as any condition that triggers and/or modifies the behaviour of a mechanism’* ^22^ (p317).

**Mechanism (M):** *‘… underlying entities, processes or structures which operate in particular contexts to generate outcomes of interest’*.^23^

**Outcome (O):** The result of the interaction between a mechanism and its triggering context

To contain the scope of the review, it initially focused only on (1) literature related to adults with type 1 diabetes and (2) the two named diabetes distress interventions under investigation in this study (TunedIn and the Danish Reduce) and the key psychological and social theories underpinning them. Thus, the research question identified for the review was:

*What aspects of the US and Danish interventions for reducing diabetes distress work, for whom, in what circumstances and why?*

The review was undertaken using a three‐stage literature searching process, incorporating stakeholder consultation throughout.

### 2.1 Stage 1: Literature directly relating to TunedIn and Danish Reduce

All study co‐applicants—including those who developed the US and Danish interventions—were asked to identify published and unpublished materials, either directly about the interventions or key papers that informed their development. We did not aim for an exhaustive search to help streamline and expedite the search process, as per rapid realist review guidelines.^18^ Inclusion criteria were that the paper must discuss type 1 diabetes, be relevant to either TunedIn or Danish Reduce and highlight evidence of any associated context, outcome or mechanism. Papers were excluded if they referred to type 2 diabetes only, were not relevant to either of the two interventions or did not highlight evidence of any associated context, outcome or mechanism. Any type of published or unpublished document was potentially applicable, including empirical research studies, literature reviews and grey literature, such as best practice guidance, presentations, and discussion pieces. Conventional quality appraisal methods were not applied but instead, each study was assessed for its ‘fitness for purpose’ by examining its relevance and rigour.^24^ In keeping with realist approaches, the focus was not on study design, but rather on whether the study offered credible, trustworthy insights that contributed to theory development.^21^, ^25^

In total, 33 potentially relevant papers were provided by the study co‐applicants. SS read all 33 papers as first reviewer and three researchers also divided the 33 papers between them as second reviewers, so that every paper was read by two researchers. A bespoke data extraction sheet was created and completed by every researcher for each paper read. Researchers met to discuss inclusion/exclusion to ensure that agreement was reached. In total, 22 papers were included in the analysis at Stage 1^2^, ^5^, ^6^, ^14^, ^15^, ^16^, ^17^, ^26^, ^27^, ^28^, ^29^, ^30^, ^31^, ^32^, ^33^, ^34^, ^35^, ^36^, ^37^, ^38^, ^39^, ^40^ and 11 were excluded (see Supplementary Material 1).

The researchers independently compiled a list of programme theories based upon the papers they had read to produce seven overarching initial programme theories and 20 ‘If‐Then‐Because’ statements as a structured way to express Context–Mechanism–Outcome configurations. The team met regularly throughout this process to explore the initial theories and statements, and all queries were discussed until agreement was reached. Engaging key stakeholders (including programme architects, adults with type 1 diabetes, diabetes practitioners and methodological experts across our co‐applicant, participant and public involvement, and co‐design groups) by presenting our initial programme theories and asking how they resonated with their personal experiences, helped us to validate our findings and identify any gaps in the literature. Realist approaches recommend this step, as understanding stakeholders' insights about the intervention and its intended purpose is vital for comprehensively analysing its implementation and impact.^41^ The overall feedback from stakeholders was that these seven initial programme theories and 20 If‐Then‐Because statements accurately captured the key ingredients of the interventions and were all valid. Some small changes were made to the theory descriptions, but no new theories were identified.

### 2.2 Stage 2: Literature relating to the psychological and social theories underpinning TunedIn and Danish Reduce

We aimed to identify whether there were any RCTs of diabetes distress‐related interventions for people with type 1 diabetes based upon the same psychological and social theories underpinning TunedIn and Danish Reduce (Table 1). We accessed a Covidence database developed for a systematic review underpinning the EASD Diabetes Distress Clinical Guidelines (Personal communication with Jackie Sturt, 9 September 2025) to identify any similar type 1 diabetes distress management interventions also underpinned by the psychological and social theories highlighted in Table 1. A total of 39 potentially relevant papers were identified from this database, but after reading all papers in full, only one was included in the review.^42^ The remainder of the papers were excluded, either because they focused on people with type 2 diabetes only, reducing diabetes distress was not an outcome of the study or they were not comparable interventions to TunedIn or Danish Reduce (i.e., not group‐based interventions delivered by a trained facilitator).

**TABLE 1:** Psychological and social theories underpinning TUNED‐IN and Danish Reduce.

Intervention	Underpinning psychological and social theories
**TUNED‐IN and Danish Reduce**	Acceptance and commitment therapy
	Self‐determination theory
	Motivational interviewing
	Empowerment‐based communication
	Peer support
	Social learning theory
	Social comparison theory

### 2.3 Stage 3: Realist reviews based upon the psychological and social theories underpinning TunedIn and Danish Reduce

A third and final stage of searching was undertaken to identify any realist reviews or realist evaluations of the psychological and social theories underpinning the TunedIn and Danish Reduce interventions in other health conditions. This is consistent with realist principles, which encourage theory testing across diverse contexts.^41^ The search was therefore widened to identify any realist reviews or evaluations based upon the psychological and social theories identified in Table 1 in any healthcare conditions involving adult participants. Expert advice was sought from Library and Information Sciences Specialists around generating relevant search terms and four electronic databases were searched on 24th February 2025 using the strategies highlighted in Box 2.

BOX 2 **Search strategy for searches in MEDLINE, Embase, HMIC and PsycINFO**

**Free text terms and operators**

*‘acceptance and commitment therapy’* OR *‘bio psycho social model’* OR *‘motivational interviewing’* OR *‘self determination theory’* OR *‘empowerment based communication’* OR *‘peer support’* OR *‘social learning theory’* OR *‘social comparison theory’*.

AND

*‘realist’*.

[**Limiters:** English language only]

After duplicates were removed, 77 papers were identified and the titles and abstracts reviewed. Sixty‐one papers were excluded due to not being realist reviews, evaluations or syntheses; being focused on children/youth; not being in a health‐related arena (e.g. education); not specifically referring to one of the psychological or social theories under investigation; or being conference abstracts only. Sixteen articles identified from the Stage 3 searches were read in full and four were included.^43^, ^44^, ^45^, ^46^ The remaining papers were excluded because they described interventions that were not relevant or comparable to TunedIn or Danish Reduce (i.e., group‐based interventions delivered by a trained facilitator).

After three stages of literature searching, 27 articles were included in this review (Supplementary Material 2). Salient details about each included paper identified in Stages 2 and 3 were recorded on the data extraction sheet and initial theories in these papers were explored as in Stage 1. Whilst supporting evidence for the theories identified in Stage 1 was found within the Stages 2 and 3 articles, no new theories were identified. The theory descriptions for the proposed ‘D‐Stress Reduce’ intervention were amended and finalised, where necessary, to include any new information gathered from the Stages 2 and 3 searches. SS then re‐read all the included papers to explore whether any specific Context–Mechanism–Outcome configurations could be identified within each theory. Context–Mechanism–Outcome configurations can be described as specific and individual causal explanations of how an intervention produces outcomes in practice. Occasionally (in some of the realist papers identified in Stage 3),^44^, ^45^ Context‐Mechanism–Outcome configurations were explicitly stated which could potentially be applicable to the ‘D‐Stress Reduce’ intervention and these were recorded. In most cases, however, retroductive reasoning was used to interpret how outcomes may have been generated by mechanisms in particular contexts. Again, these Context–Mechanism–Outcome configurations were circulated amongst the study stakeholders and modifications were made, where required. One key suggestion from our methodological expert was that the Context–Mechanism–Outcome configurations be worded as ‘If‐Then‐Because’ statements, to allow for tentative theory building without needing to rigidly assign elements to ‘Context’, ‘Mechanism’ or ‘Outcome’ categories. These statements helped to articulate tentative causal pathways and provide scaffolding for further Context–Mechanism–Outcome configuration development in the subsequent realist process evaluation.

## 3 RESULTS

Twenty‐seven papers, published between 2000 and 2024, were included and comprised published research articles (*n* = 13); PowerPoint presentations/training materials (*n* = 6); published reviews (*n* = 5); discussion pieces (*n* = 2) and an unpublished abstract. The papers referred to work undertaken primarily in the US (*n* = 17), followed by Denmark (*n* = 5), the UK (*n* = 2), Germany (*n* = 1), Australia (*n* = 1) and The Netherlands (*n* = 1). Fourteen were primarily thought to be relevant to TunedIn and eight to the Danish Reduce. The remaining papers were picked up in Stages 2 and 3 of the study and were related to the psychological and social theories underpinning them.

**FIGURE 2:**
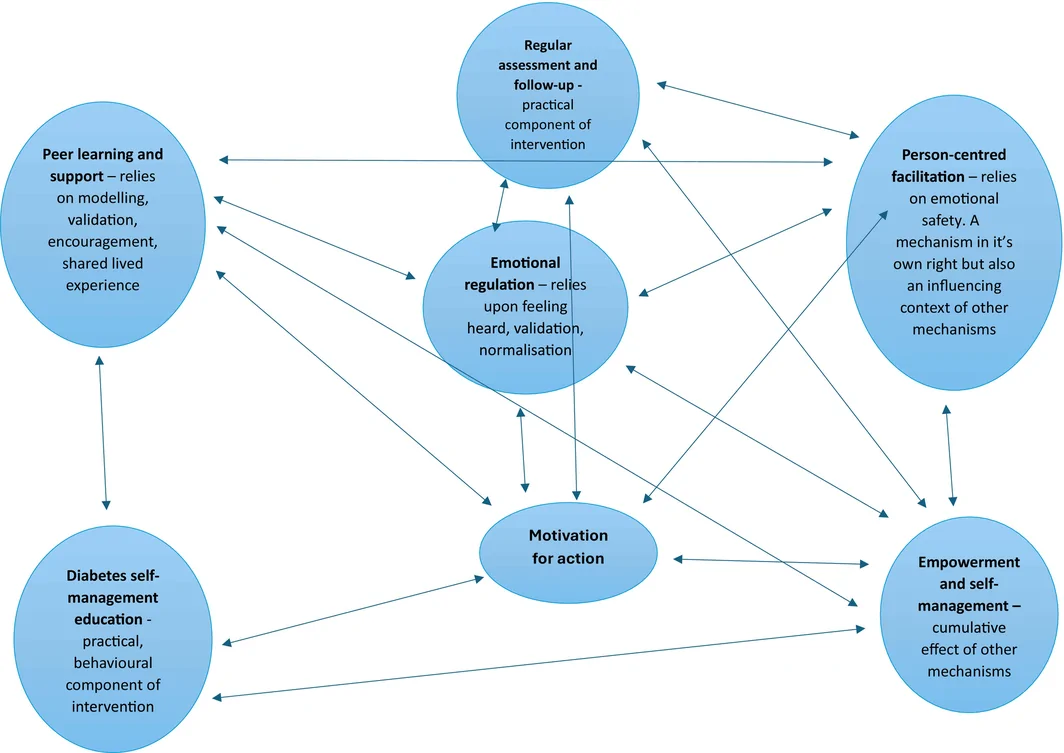
Visual representation of interlapping relationships between programme theories.

Seven programme theories were developed, and each was supported by evidence from the literature and articulated through ‘If‐Then‐Because’ statements to illustrate causal pathways. A selection of If‐Then‐Because statements are highlighted in Boxes 3, 4, 5, 6, 7, 8 and 9, alongside illustrative quotes from the literature, where available. All 20 If‐Then‐Because statements are described in Table 2. A visual example of how programme theories might map into their contextual factors and outcomes is provided in Supplementary Material 3. A visual representation of the interlapping relationships between programme theories is provided in Figure 2.

**TABLE 2:** Context–mechanism–outcome configurations for the D‐Stress Reduce intervention.

Number	Context–mechanism–outcome configuration description
1	**IF** an adult with type 1 diabetes has not previously had the opportunity to talk about their emotions or is unable to articulate how they feel, **THEN** they gain greater emotional clarity **BECAUSE** the intervention teaches them to identify, label, and verbalise previously unspoken feelings
2	**IF** an adult with type 1 diabetes is not aware of how they act when experiencing negative emotions or has developed avoidant/reactive behaviours associated with these emotions, **THEN** the intervention empowers them to gain a greater sense of control over negative emotions **BECAUSE** it teaches them to accept and manage negative emotions rather than avoid or react to them
3	**IF** an adult with type 1 diabetes has feelings of guilt or shame surrounding their type 1 diabetes, **THEN** the intervention helps them to adopt more compassionate self‐assessments and reduce their feelings of self‐criticism **BECAUSE** it encourages them to challenge or reframe critical thoughts and focus on more self‐compassionate goals or expectations
4	**IF** the group facilitators foster a safe space for sharing emotions by modelling empathy and mutual respect, **THEN** adults with type 1 diabetes are more likely to explore their own feelings, beliefs, stories, and expectations **BECAUSE** a sense of safety encourages vulnerability, honesty, and empathy
5	**IF** the group does not have effective facilitation, **THEN** adults with type 1 diabetes may experience reduced emotional well‐being, anxiety over own recovery or sadness over others' suffering **BECAUSE** individuals may be interrupted or not have time to speak, peers may focus on sharing only negative feelings and experiences or may not comfort each other when distressed
6	**IF** adults with type 1 diabetes believe that healthcare professionals have limited knowledge, understanding or time to explore diabetes distress **THEN** they are more likely to reject outside professional advice and take on board peer suggestions **BECAUSE** peer support is perceived as a more credible, safer or better option
7	**IF** an adult with type 1 diabetes feels isolated in their diabetes distress and the intervention provides regular peer interaction **THEN** they feel less alone **BECAUSE** shared experiences foster validation and connection
8	**IF** adults with type 1 diabetes have low social confidence, **THEN** they may experience anxiety about the intervention ending and the associated loss of social support **BECAUSE** whilst the intervention may operate as a safe bubble for an individual, they may remain unable to move beyond it to develop authentic relationships with non‐peers
9	**IF** adults with type 1 diabetes compare their experiences of diabetes distress to their peers, **THEN** they can either experience greater levels of self‐compassion and relief or feel more self‐critical and alone **BECAUSE** comparing experiences can either help normalise their distress (if peers experience similar emotions) or increase it (if they do not)
10	**IF** adults with type 1 diabetes are exposed to peers with diverse backgrounds, needs, skills, and experiences **THEN** they are better able to embrace their own unique challenges and strengths and handle their diabetes distress in their own way **BECAUSE** they gain a broader understanding of individualised approaches to managing diabetes distress.
11	**IF** there is limited access to one‐to‐one mental health support locally and an adult with type 1 diabetes wants, but is unable to access, one‐to‐one counselling **THEN** they may be dissatisfied with their experience of the intervention **BECAUSE** they may view group support as inferior to one‐to‐one support or perceive the intervention as a holding position whilst waiting for counselling
12	**IF** an adult with type 1 diabetes does not want to talk about themselves and the group is poorly attended **THEN** they may feel a sense of discomfort during the sessions **BECAUSE** they feel overly pressured to talk
13	**IF** group facilitators adopt a collaborative rather than authoritative role **THEN** people feel more heard and respected **BECAUSE** this approach fosters trust and shared responsibility
14	**IF** adults with type 1 diabetes with low baseline emotional regulation skills request self‐management education **THEN** they may feel confused and overwhelmed by this information, potentially increasing their diabetes distress **BECAUSE** they can find it difficult to process new information and apply it to their own lives
15	**IF** adults with type 1 diabetes with strong emotional regulation skills request self‐management education, **THEN** they improve their ability to manage type 1 diabetes **BECAUSE** they can absorb and apply new knowledge effectively
16	**IF** diabetes distress is assessed and followed up regularly **THEN** adults with type 1 diabetes gain clarity and focus on managing it **BECAUSE** ongoing dialogue reinforces diabetes distress as a continuous care priority
17	**IF** the intervention provides adults with type 1 diabetes with three primary psychological needs of competence, relatedness, and autonomy, **THEN** they are more likely to experience sustained behavioural change, greater well‐being and self‐esteem **BECAUSE** these three needs foster autonomous (intrinsic) motivation, leading to long‐term engagement
18	**IF** group facilitators assess and respond to individual motivation levels **THEN** adults with type 1 diabetes are more likely to engage **BECAUSE** personalised support promotes self‐direction and readiness for change
19	**IF** the intervention emphasises adults with type 1 diabetes as the primary decision makers in their lives **THEN** this fosters a sense of control and self‐efficacy, leading to a more positive outlook on their ability to manage their diabetes distress **BECAUSE** people experience a shift in mindset, recognising that they are the experts in their own lives
20	**IF** the intervention encourages adults with type 1 diabetes to set their own goals and these differ from those prioritised by healthcare professionals, **THEN** this creates tension in the collaborative care partnership **BECAUSE** differing priorities can challenge the balance between empowerment and clinical guidance

### 3.1 Programme theory 1: Emotional regulation (*n* = 15)

Fifteen articles addressed the theory of emotional regulation,^14^, ^15^, ^16^, ^17^, ^27^, ^29^, ^30^, ^33^, ^34^, ^36^, ^37^, ^38^, ^39^, ^40^, ^42^ highlighting how developing emotional regulation skills can play a central role in reducing diabetes distress. Emotional regulation can be defined as the effort an individual makes (implicitly or explicitly) to influence the experience, expression and effects of emotions. These may include awareness, mindfulness, clarity, acceptance and understanding of emotion, self‐compassion and self‐empathy, and a readiness and ability to act in accordance with a plan.^14^, ^27^ This theory centres on how some people may not have the vocabulary, skills or opportunity to articulate their emotional experiences of living with diabetes. Some individuals may also experience self‐criticism about their diabetes rooted in guilt and shame, whilst others may internalise criticism or blame from those around them, and this may prevent them from discussing their distress. This evidence suggests that a diabetes distress intervention should seek to foster emotional regulation through the development of self‐awareness and self‐compassion, aiming to normalise and validate emotional experiences. Articulating previously unspoken fears and frustrations will help individuals gain emotional clarity and a deeper understanding of their reactions, laying the groundwork for behavioural change. Contextual factors that might influence this theory include the persons' age, number of diabetes‐related complications, and whether they perceive their environment as emotionally safe. Working alongside a warm and empathetic group facilitator may also enhance a persons' ability to recognise and reflect upon their emotions.

BOX 3 **Programme theory 1: Emotional regulation**

**Example Context–Mechanism–Outcome configuration: IF** an adult with type 1 diabetes has not previously had the opportunity to talk about their emotions or is unable to articulate how they feel **THEN** they gain greater emotional clarity **BECAUSE** the intervention teaches them to identify, label, and verbalise previously unspoken feelings.

**Example quote:** *‘As time passed I was much better at sharing my feelings (…), suddenly I thought “wow, I can do this”’*.^16^

### 3.2 Programme theory 2: Peer learning and support (*n* = 11)

Eleven papers addressed the theory of peer learning and support,^14^, ^16^, ^17^, ^26^, ^29^, ^34^, ^36^, ^43^, ^44^, ^45^, ^46^ highlighting the powerful role of peer connection in influencing change amongst adults with type 1 diabetes. This theory centres on the idea that many adults with type 1 diabetes feel less alone and more inspired to reflect or act upon their diabetes distress when hearing the first‐hand experiences and challenges of their peers, as opposed to receiving advice from healthcare professionals. Despite a desire to connect, many people with type 1 diabetes experience feelings of isolation and lack opportunities to meet others who share similar experiences with diabetes distress. This evidence suggests that a diabetes distress intervention should create safe, inclusive opportunities for adults with type 1 diabetes to share and learn from one another's lived experiences, harnessing the motivating and normalising power of peer connection to reduce diabetes distress. However, the literature also suggests the group experience will not be universally positive. Some individuals may feel increased isolation if they perceive themselves to be too different from others, anxious after hearing about others' complications, or believe they are not managing their diabetes as well as their peers.^34^ Thus, the peer‐based approach can be shaped by several contextual factors, including the skill and sensitivity of the group facilitator, how safe people feel within the group and the diversity of the group members. Variations among participants may include factors such as age, educational background, reason for joining, and level of need for peer support.

BOX 4 **Programme theory 2: Peer learning and support**

**Example Context–Mechanism–Outcome configuration: IF** an adult with type 1 diabetes feels isolated in their diabetes distress and the intervention provides regular peer interaction **THEN** they feel less alone **BECAUSE** shared experiences foster validation and connection.

**Example quote:** ‘*Meeting others in the same situation or who have been in the same situation, others with the same experiences, the same anxiety, the same crises, and the same kind of loneliness… That was very important’*.^26^

### 3.3 Programme theory 3: Person‐centered facilitation (*n* = 8)

Eight papers addressed changing healthcare professionals' usual ways of interacting with adults with type 1 diabetes^6^, ^17^, ^28^, ^29^, ^31^, ^32^, ^36^, ^46^ as this often follows a hierarchical model, where clinicians may dominate the conversation, interrupt the person with type 1 diabetes or adopt the role of ‘fixer’. This evidence suggests that a diabetes distress intervention should support group facilitators to move away from hierarchical, directive styles of communication and towards collaborative, person‐centred interactions. The facilitator focuses on understanding and supporting the emotional experiences of participants, rather than delivering information or offering solutions. By fostering trust, respect, and shared responsibility, such an approach can empower adults with type 1 diabetes to feel more valued in their care and more engaged in addressing their diabetes distress. This paradigm shift may be challenging for some group facilitators, particularly those used to being seen as the authority figure or who feel discomfort with less structured conversations. Training, reflection, and ongoing support will therefore be required for group facilitators delivering this intervention.

BOX 5 **Programme theory 3: Person‐centered facilitation**

**Example Context–Mechanism–Outcome configuration: IF** group facilitators adopt a collaborative rather than authoritative role **THEN** people feel more heard and respected **BECAUSE** this approach fosters trust and shared responsibility.

### 3.4 Programme theory 4: Diabetes self‐management education (*n* = 7)

Seven articles addressed diabetes self‐management education.^5^, ^14^, ^15^, ^30^, ^33^, ^34^, ^36^ Although this will not be a core component of the D‐Stress Reduce intervention, the literature highlighted that people with type 1 diabetes might express a desire for this type of support, or these conversations may emerge organically within group discussions. These moments of self‐management education reflect a mechanism identified (particularly in the Danish Reduce) as important for enhancing motivation, self‐efficacy, and connection. This highlights the importance of integrated delivery, where education and practical management insights can complement emotional reflection without overwhelming the intervention's primary focus. This flexibility to explore self‐management education where requested may particularly benefit people with type 1 diabetes who already have strong emotional regulation skills and lower distress, as they may be more able to absorb and apply new knowledge.^15^ Although diabetes self‐management education will not be a core component, D‐Stress Reduce should equip group facilitators to recognise when such needs arise and to respond in a balanced way that supports individuals' requests without detracting from the intervention's primary aims.

BOX 6 **Programme theory 4: Diabetes self‐management education**

**Example Context–Mechanism–Outcome configuration: IF** adults with type 1 diabetes with strong emotional regulation skills request self‐management education, **THEN** they improve their ability to manage type 1 diabetes **BECAUSE** they can absorb and apply new knowledge effectively.

**Example quote:** *‘A very concrete thing was how to place the sensors and the insulin. Many peers said they started at the back and then took it all the way around, and then opposite again. I actually found it interesting to hear and I've adopted this’*.^34^

### 3.5 Programme theory 5: Regular assessment and follow‐up (*n* = 6)

Six papers addressed the theory of regular assessment and follow‐up,^14^, ^28^, ^29^, ^36^, ^39^, ^47^ highlighting how people with type 1 diabetes are often not provided with a consistent, structured space to verbalise their diabetes distress or receive systematic follow‐up after these conversations. This lack of continuity means that emotional concerns are treated as isolated issues rather than part of ongoing care and leaves individuals without the opportunity to receive appropriate emotional support and engage in collaborative action planning for behavioural change. The literature suggests that an ongoing, structured approach to exploring diabetes distress not only normalises these discussions but also signals that emotional well‐being is a legitimate and integral component of diabetes management. This can help pave the way for meaningful behavioural change by encouraging value‐based action planning, enabling people to identify what matters to them and to set goals aligned with their personal values.

BOX 7 **Programme theory 5: Regular assessment and follow‐up**

**Example Context–Mechanism–Outcome configuration: IF** diabetes distress is assessed and followed up regularly **THEN** adults with type 1 diabetes gain clarity and focus on managing it **BECAUSE** ongoing dialogue reinforces diabetes distress as a continuous care priority.

**Example quote:** *‘After the* [group sessions], *I have begun using that app* [from the Danish Diabetes Association]. *That is a very concrete example of something I have done afterward’*.^34^

### 3.6 Programme theory 6: Motivation for action (*n* = 4)

Four papers addressed the theory of motivation for action,^28^, ^34^, ^35^, ^46^ which highlights how motivation for change in people with type 1 diabetes varies both between individuals and within the same person across different behaviours. This variability makes it essential for successful interventions to assess and respond to individual motivation levels thoughtfully within each session and for each type of change. There was some evidence to suggest that successful interventions are required to meet three key psychological needs: competence (feeling capable and confident), relatedness (feeling understood and supported), and autonomy (having control and ownership over one's actions). When these needs are nurtured, people are more likely to feel internally motivated, leading to greater self‐regulation and enhanced well‐being.^35^ Facilitators can promote internal motivation by avoiding putting pressure, imposed goals, or external rewards onto participants and instead focusing on fostering self‐direction and personal choice.

BOX 8 **Programme theory 6: Motivation for action**

**Example Context–Mechanism–Outcome configuration: IF** group facilitators assess and respond to individual motivation levels **THEN** adults with type 1 diabetes are more likely to engage **BECAUSE** personalised support promotes self‐direction and readiness for change.

**Example quote**: *‘I have felt motivated to do something about blood glucose measurements. Many doctors have tried to motivate me. I think it has been much more motivating to be part of this group’*.^26^

### 3.7 Programme theory 7: Empowerment and self‐management (*n* = 2)

Finally, two papers addressed adults with type 1 diabetes' perceptions of healthcare and personal responsibility for behaviour change.^17^, ^32^ This represents a complementary process to the mindset shift required by healthcare professionals (programme theory 3) and highlights the interdependence between person empowerment and person‐centred facilitation. The evidence suggests that successful interventions help adults with type 1 diabetes to become active partners in their own care, enabling them to discover and develop their inherent capacity to take control and make primary decisions in their lives. This empowerment may lead to improved problem‐solving, better communication with healthcare professionals outside of the D‐Stress Reduce setting, increased satisfaction with care, and greater confidence in their ability to manage their diabetes distress.

BOX 9 **Programme theory 7: Empowerment and self‐management**

**Example Context–Mechanism–Outcome configuration: IF** the intervention emphasises adults with type 1 diabetes as the primary decision makers in their lives **THEN** this fosters a sense of control and self‐efficacy, leading to a more positive outlook on their ability to manage their diabetes distress **BECAUSE** people experience a shift in mindset, recognising that they are the experts in their own lives.

**Example Context–Mechanism–Outcome configuration 2: IF** the intervention encourages adults with type 1 diabetes to set their own goals and these differ from those prioritised by healthcare professionals, **THEN** this creates tension in the collaborative care partnership **BECAUSE** differing priorities can challenge the balance between empowerment and clinical guidance.

## 4 DISCUSSION

This rapid realist review aimed to generate context‐sensitive, theory‐driven programme theories around for whom, how and why a D‐Stress Reduce intervention might reduce elevated diabetes distress in adults with type 1 diabetes in the UK healthcare context. As D‐Stress Reduce will be based upon two existing diabetes distress interventions—TunedIn (US) and Reduce (Denmark)—evidence from these programmes was synthesised to identify the mechanisms through which they were thought to reduce elevated diabetes distress, the outcomes they could produce and the contexts which might influence how they work. The review identified several complementary theories that could underpin the D‐Stress Reduce intervention, although there was more evidence for some than others. Notably, emotional regulation was identified as both a target for intervention and a mechanism for change in over half the papers. This is consistent with previous research showing that individuals with type 1 diabetes often lack opportunities to reflect upon or articulate their emotional experiences of living with diabetes.^6^, ^10^, ^48^

Peer support and learning also emerged as another key mechanism, highlighting the potential for D‐Stress Reduce to reduce isolation and normalise emotional experiences through shared narratives and connections. The review suggested that hearing the lived experiences of others with type 1 diabetes may be more beneficial than receiving advice from healthcare professionals or facilitators—a finding consistent with broader evidence on the value of peer support in chronic illness management.^49^ The intervention needs to remain mindful however that the group dynamic will not be universally positive; for some adults with type 1 diabetes, comparison with others may increase distress. Peer support interventions for managing elevated diabetes distress do not have a strong RCT evidence base despite considerable observational evidence. Such experimental studies are important for establishing the outcomes on diabetes distress of peer support.^50^, ^51^ Our findings underscore the importance of appropriately trained and skilled group facilitators who can ensure constructive, inclusive, and emotionally safe peer interactions. This review has further highlighted the importance of group facilitators maintaining a person‐centred, collaborative approach, engaging in active listening using open‐ended questions and allowing silences. Creating a space where adults with type 1 diabetes can feel heard and respected will aim to support a more equal and reflective dialogue.

In comparison, evidence was more limited for some programme theories in the review, including motivation for action. This may suggest that this mechanism is less pivotal to the intervention success *or* that existing studies have not examined it in sufficient depth. Although there was limited evidence for some programme theories, all seven warrant closer attention to explore how they unfold in practice and whether they represent meaningful ways to reduce diabetes distress for adults with type 1 diabetes. Whilst this review aimed to directly inform the delivery of ‘Reduce’ in the UK, evaluation of emotional regulation for managing elevated diabetes distress in other intervention modalities and contexts is warranted, such as with marginalised communities and with different cultures where emotional distress can be perceived as a spiritual, as opposed to a psychological, need.^52^ New programme theories not identified in this rapid realist review may also emerge in the subsequent realist process evaluation, highlighting additional factors that are necessary for the success of diabetes distress interventions in the UK healthcare context. Remaining open to the development of such new theories will be essential to ensure that the process evaluation captures the full complexity of diabetes distress and identifies mechanisms that might otherwise remain overlooked.

Beyond informing the co‐adaptation of the D‐Stress Reduce intervention, this review provides broader understanding of diabetes distress by clarifying the mechanisms through which group‐based psychosocial interventions exert their effects. The programme theories generated, particularly those relating to emotional regulation, peer learning, and shifts in healthcare communication, offer broader insights into the psychological and relational processes that influence diabetes distress across settings. Furthermore, these findings have relevance for the design of other psychosocial interventions beyond the D‐Stress programme. By identifying mechanisms that appear to operate across different healthcare systems and delivery models, the review highlights the factors, such as emotional safety, facilitator stance, and opportunities for peer validation, that may represent core components in diabetes distress support more broadly.

Methodologically, this study demonstrates the value of realist syntheses for investigating complex psychosocial interventions in diabetes care. The realist approach enabled us to move beyond questions of *‘what works’* to illuminate *how* and *why* interventions work and for whom. This has broader implications for a growing methodological agenda that requires theory‐driven evidence synthesis in behavioural diabetes research. Future research is needed to build upon these programme theories through study designs capable of testing both effectiveness and implementation processes. Hybrid effectiveness–implementation designs, realist process evaluations, and longitudinal mixed‐methods studies should allow researchers to examine not only whether diabetes distress decreases but whether mechanisms, such as emotional regulation, peer connection, and facilitator communication styles change over time. There is also a need to explore how these mechanisms operate in under‐researched populations and contexts, including marginalised groups, culturally diverse communities, and healthcare systems with resource constraints. Understanding these contextual variations is essential for refining diabetes distress interventions internationally to meet local need. Finally, future evaluations should incorporate outcomes aligned with the mechanisms identified in this review, such as emotional safety, engagement, and healthcare relationships, as these outcomes may provide more sensitive indicators of change than glycaemic measures alone.

### 4.1 Strengths and limitations

Using a realist approach to explore the literature associated with TunedIn and the Danish Reduce interventions enabled the authors to highlight the causal explanations and contextual factors often overlooked in traditional systematic reviews. Stakeholder consultation further enhanced the credibility and relevance of the findings, ensuring that the theories resonated with lived experience. The authors acknowledge that they did not aim for an exhaustive search as per rapid realist review guidelines. The literature also came mainly from Western, high‐income countries, such as the US and Denmark, meaning the findings of this review may not be relevant to all countries. However, this review represents the first stage of a wider realist process evaluation, in which theories will be further tested and refined. The purpose of this review was to help guide the development of a new co‐adapted intervention in a UK setting. Finally, this review focused only on reducing diabetes distress in adults with type 1 diabetes, which is an additional limitation. Although several of the theories identified—particularly those relating to emotional regulation, peer support, and person‐centred care—are likely relevant to adults with type 2 diabetes, the contextual factors influencing distress may differ substantially.^10^ Future research should assess whether and how these theories might translate for adults with type 2 diabetes.

## 5 CONCLUSION

This review suggests that the new D‐Stress Reduce intervention should likely operate through multiple, interacting mechanisms rather than a single causal pathway. Its effectiveness will depend on individual needs, experiences and the context in which it is delivered, highlighting the complexity of addressing elevated diabetes distress and the importance of a multilayered, context‐sensitive intervention. Future evaluation will need to examine how these contexts, mechanisms and outcomes interact in practice and adapt to the diverse circumstances of adults with type 1 diabetes. Whilst strengthening the RCT evidence base for some components such as emotional regulation and peer support, our review indicates the importance of hybrid trial designs^53^ which evaluate context and the proposed programme theories on outcomes alongside an experimental focus.

## FUNDING INFORMATION

This realist review was funded as part of the D‐Stress study by a grant to Jackie Sturt (King's College London, UK) from the National Institute for Health and Care Research and Diabetes UK (NIHR205441). Jane Speight is supported by the core funding to the Australian Centre for Behavioural Research in Diabetes, provided by the collaboration between Diabetes Victoria and Deakin University, Australia. Siobhan O'Connor is supported through an NIHR Development and Skills Enhancement Award (NIHR305124).

## CONFLICT OF INTEREST STATEMENT

Richard Holt and Jane Speight are the co‐chairs, and Jackie Sturt is a panel member of the 2026 EASD evidence‐based clinical practice guideline for assessing and managing diabetes distress among adults with type 1 diabetes and type 2 diabetes.

## Supporting information


Supplementary Material 1.

Supplementary Material 2.

Supplementary Material 3.

Supplementary Material 4.

## Data Availability

The data that support the findings of this study are available from the corresponding author upon reasonable request.
