# Supplementary material for: Group‐based interventions to reduce diabetes distress in adults with type 1 diabetes: A rapid realist review of the Reduce and TunedIn interventions
Source: Diabet Med. 2026 Feb 12;43(9):e70242. doi: 10.1111/dme.70242 (PMC13614002; doi:10.1111/dme.70242)
Supplement: Supplementary file 1 [file DME-43-e70242-s001.docx]

**Supplementary material 1 - Papers excluded in the rapid realist review**

Chew BH, Vos RC, Stellato RK, et al. The effectiveness of an emotion-focused educational programme in reducing diabetes distress in adults with Type 2 diabetes mellitus (VEMOFIT): a cluster randomized controlled trial. *Diabet Med* 2018;35(6):750-59.

Chew BH, Vos RC, Fernandez A, et al. The effectiveness of an emotion-focused educational programme in reducing diabetes distress in adults with type 2 diabetes mellitus at 12-month follow-up: a cluster randomized controlled trial. *Ther Adv Endocrinol Metab* 2019;10:2042018819853761.

Fisher L, Mullan JT, Arean P, et al. Diabetes distress but not clinical depression or depressive symptoms is associated with glycemic control in both cross-sectional and longitudinal analyses. *Diabetes Care* 2010;33(1):23-8.

Fisher L, Hessler DM, Polonsky WH, et al. When is diabetes distress clinically meaningful?: establishing cut points for the Diabetes Distress Scale. *Diabetes Care* 2012;35(2):259-64.

Fisher L, Polonsky WH, Hessler DM, et al. Understanding the sources of diabetes distress in adults with type 1 diabetes. *J Diabetes Complications* 2015;29(4):572-7.

Fisher L, Hessler D, Polonsky W, et al. Diabetes distress in adults with type 1 diabetes: Prevalence, incidence and change over time. *J Diabetes Complications* 2016;30(6):1123-8.

Nygaard M, Willaing I, Joensen LE, et al. A Short-Form Measure of Diabetes Distress Among Adults With Type 1 Diabetes for Use in Clinical Practice: Development and Validation of the T1-DDS-7. *Diabetes Care* 2023;46(9):1619-25.

Polonsky WH, Anderson BJ, Lohrer PA, et al. Assessment of diabetes-related distress. *Diabetes Care* 1995;18(6):754-60.

Stenov V, Wind G, Skinner T, et al. The potential of a self-assessment tool to identify healthcare professionals’ strengths and areas in need of professional development to aid effective facilitation of group-based, person-centered diabetes education. *BMC Medical Education* 2017;17.

Stenov V, Christensen J, Due-Christensen M, et al. 655-P: Realizing the Hidden Emotional Burden—Systematic Detection of Diabetes Distress in Adults with Type 1 Diabetes in Routine Consultations. *Diabetes* 2024c;73 (Supplement 1).

TUNED-IN-course-materials-5. TUNED-I Moving Forward. Unpublished.

Bendig E, Bauereiss N, Schmitt A, et al. ACTonDiabetes—a guided psychological internet intervention based on Acceptance and Commitment Therapy (ACT) for adults living with type 1 or 2 diabetes: results of a randomised controlled feasibility trial. *BMJ Open* 2021;11(7):e049238.

Brennan MC, Albrecht MA, Brown JA, et al. Self-Management Group Education to Reduce Fear of Hypoglycemia as a Barrier to Physical Activity in Adults Living With Type 1 Diabetes: A Pilot Randomized Controlled Trial. *Canadian Journal of Diabetes* 2021;45(7):619-28.

Chan JCN, Sui Y, Oldenburg B, et al. Effects of Telephone-Based Peer Support in Patients With Type 2 Diabetes Mellitus Receiving Integrated Care: A Randomized Clinical Trial. *JAMA Internal Medicine* 2014;174(6):972-81.

Cornely RM, Subramanya V, Owen A, et al. A mixed-methods approach to understanding the perspectives, experiences, and attitudes of a culturally tailored cognitive behavioral therapy/motivational interviewing intervention for African American patients with type 2 diabetes: a randomized parallel design pilot study. *Pilot and Feasibility Studies* 2022;8(1):107.

Dale J, Caramlau I, Sturt J, et al. Telephone peer-delivered intervention for diabetes motivation and support: The telecare exploratory RCT. *Patient Education and Counseling* 2009;75(1):91-98.

Ewen AM, Hawkins JM, Kloss KA, et al. The Michigan Men’s Diabetes Project Randomized Clinical Control Trial: A Pilot/Feasibility Study of a Peer-Led Diabetes Self-Management and Support Intervention for Black Men With Type 2 Diabetes. *American Journal of Men's Health* 2024;18(3):15579883241258318.

Gabbay RA, Añel-Tiangco RM, Dellasega C, et al. Diabetes nurse case management and motivational interviewing for change (DYNAMIC): Results of a 2-year randomized controlled pragmatic trial, *Journal of Diabetes* 2013;5(3):349-57.

Gagliardino JJ, Arrechea V, Assad D, et al. Type 2 diabetes patients educated by other patients perform at least as well as patients trained by professionals. *Diabetes/Metabolism Research and Reviews* 2013;29(2):152-60.

Glasgow RE, Nutting PA, King DK, et al. A practical randomized trial to improve diabetes care. *Journal of General Internal Medicine* 2004;19(12):1167-74.

Griva K, Rajeswari M, Nandakumar M, et al. The combined diabetes and renal control trial (C-DIRECT) - a feasibility randomised controlled trial to evaluate outcomes in multi-morbid patients with diabetes and on dialysis using a mixed methods approach. *BMC Nephrology* 2019;20(1):2.

Heinrich E, Candel MJJM, Schaper NC, et al. Effect evaluation of a Motivational Interviewing based counselling strategy in diabetes care. *Diabetes Research and Clinical Practice* 2010;90(3):270-78.

Heisler M, Vijan S, Makki F, et al. Diabetes Control With Reciprocal Peer Support Versus Nurse Care Management. *Annals of Internal Medicine* 2010;153(8):507-15.

Hoyo MLLd, Rodrigo MTF, Urcola-Pardo F, et al. The TELE-DD Randomised Controlled Trial on Treatment Adherence in Patients with Type 2 Diabetes and Comorbid Depression: Clinical Outcomes after 18-Month Follow-Up. *International Journal of Environmental Research and Public Health* 2023;20(1):328.

Ismail K, Winkley K, de Zoysa N, et al. Nurse-led psychological intervention for type 2 diabetes: a cluster randomised controlled trial (Diabetes-6 study) in primary care. *British Journal of General Practice* 2018;68(673):e531.

Ju C, Shi R, Yao L, et al. Effect of peer support on diabetes distress: a cluster randomized controlled trial. *Diabetic Medicine* 2018;35(6):770-75.

Juul L, Maindal HT, Zoffmann V, et al. Effectiveness of a Training Course for General Practice Nurses in Motivation Support in Type 2 Diabetes Care: A Cluster-Randomised Trial. *PLOS ONE* 2014;9(5):e96683.

Kılıç A, Hudson J, Scott W, et al. An online acceptance, commitment, and self-compassion based treatment to decrease psychological distress in people with type 2 diabetes: A feasibility randomised-controlled trial. *Internet Interventions* 2023;33:100658.

Kunik ME, Evans TL, Christie IC, et al. The impact of veteran support and resources for diabetes (iNSPiRED) on diabetes distress: Results from a randomized, parallel-group trial. *General Hospital Psychiatry* 2023;85:55-62.

Kwan BM, Dickinson LM, Dailey-Vail J, et al. Comparative Effectiveness of Patient-Driven versus Standardized Diabetes Shared Medical Appointments: A Pragmatic Cluster Randomized Trial. *Journal of General Internal Medicine* 2024;39(15):2970-79.

Li Z, Chen Q, Yan J, et al. Effectiveness of motivational interviewing on improving Care for Patients with type 2 diabetes in China: A randomized controlled trial. *BMC Health Services Research* 2020;20(1):57.

Liu Y, Han Y, Shi J, et al. Effect of peer education on self-management and psychological status in type 2 diabetes patients with emotional disorders. *Journal of Diabetes Investigation* 2015;6(4):479-86.

Maghsoudi Z, Razavi Z, Razavi M, et al. Efficacy Of Acceptance And Commitment Therapy For Emotional Distress In The Elderly With Type 2 Diabetes: A Randomized Controlled Trial. *Diabetes Metab Syndr Obes* 2019;12:2137-43.

Mitchell SE, Bragg A, De La Cruz BA, et al. Effectiveness of an Immersive Telemedicine Platform for Delivering Diabetes Medical Group Visits for African American, Black and Hispanic, or Latina Women With Uncontrolled Diabetes: The Women in Control 2.0 Noninferiority Randomized Clinical Trial. *J Med Internet Res* 2023;25:e43669.

Ngan HY, Chong YY, Loo KM, et al. Preliminary efficacy of an acceptance-based diabetes education (ACT-DE) programme for people with type 2 diabetes on diabetes distress and self-care behaviours: A pilot randomised controlled trial. *Journal of Contextual Behavioral Science* 2023;30:50-60.

Piatt GA, Rodgers EA, Xue L, et al. Integration and Utilization of Peer Leaders for Diabetes Self-Management Support: Results From Project SEED (Support, Education, and Evaluation in Diabetes). *The Diabetes Educator* 2018;44(4):373-82.

Presley C, Agne A, Shelton T, et al. Mobile-Enhanced Peer Support for African Americans with Type 2 Diabetes: a Randomized Controlled Trial. *Journal of General Internal Medicine* 2020;35(10):2889-96.

Ramallo-Fariña Y, Rivero-Santana A, García-Pérez L, et al. Patient-reported outcome measures for knowledge transfer and behaviour modification interventions in type 2 diabetes—the INDICA study: a multiarm cluster randomised controlled trial. *BMJ Open* 2021;11(12):e050804.

Rosenbek Minet LK, Wagner L, Lønvig EM, et al. The effect of motivational interviewing on glycaemic control and perceived competence of diabetes self-management in patients with type 1 and type 2 diabetes mellitus after attending a group education programme: a randomised controlled trial. *Diabetologia* 2011;54(7):1620-29.

Safford MM, Andreae S, Cherrington AL, et al. Peer Coaches to Improve Diabetes Outcomes in Rural Alabama: A Cluster Randomized Trial. *The Annals of Family Medicine* 2015;13(Suppl 1):S18.

Siminerio L, Ruppert KM, Gabbay RA. Who Can Provide Diabetes Self-Management Support in Primary Care?:Findings From a Randomized Controlled Trial. *The Diabetes Educator* 2013;39(5):705-13.

Simmons D, Prevost AT, Bunn C, et al. Impact of community based peer support in type 2 diabetes: a cluster randomised controlled trial of individual and/or group approaches. *PLoS One* 2015;10(3):e0120277.

Spencer MS, Kieffer EC, Sinco B, et al. Outcomes at 18 Months From a Community Health Worker and Peer Leader Diabetes Self-Management Program for Latino Adults. *Diabetes Care* 2018;41(7):1414-22.

Swoboda CM, Miller CK, Wills CE. Impact of a goal setting and decision support telephone coaching intervention on diet, psychosocial, and decision outcomes among people with type 2 diabetes. *Patient Education and Counseling* 2017;100(7):1367-73.

Tang TS, Afshar R, Elliott T, et al. From clinic to community: A randomized controlled trial of a peer support model for adults with type 2 diabetes from specialty care settings in British Columbia. *Diabetic Medicine* 2022;39(11):e14931.

Tang TS, Funnell M, Sinco B, et al. Comparative Effectiveness of Peer Leaders and Community Health Workers in Diabetes Self-management Support: Results of a Randomized Controlled Trial. *Diabetes Care* 2014;37(6):1525-34.

Tang TS, Funnell MM, Sinco B, et al. Peer-Led, Empowerment-Based Approach to Self-Management Efforts in Diabetes (PLEASED): A Randomized Controlled Trial in an African American Community. *The Annals of Family Medicine* 2015;13(Suppl 1):S27.

Welch G, Zagarins SE, Feinberg RG, et al. Motivational interviewing delivered by diabetes educators: Does it improve blood glucose control among poorly controlled type 2 diabetes patients? *Diabetes Research and Clinical Practice* 2011;91(1):54-60.

Wijk I, Amsberg S, Johansson U-B, et al. Impact of an Acceptance and Commitment Therapy programme on HbA1c, self-management and psychosocial factors in adults with type 1 diabetes and elevated HbA1c levels: a randomised controlled trial. *BMJ Open* 2023;13(12):e072061.

Woodard L, Amspoker AB, Hundt NE, et al. Comparison of Collaborative Goal Setting With Enhanced Education for Managing Diabetes-Associated Distress and Hemoglobin A1c Levels: A Randomized Clinical Trial. *JAMA Network Open* 2022;5(5):e229975-e75.

Bardon C, Guillemette A, Rioux M-E, et al. Group intervention programs and their impact on well-being and quality-of-life for adults living with a rare or orphan disease - Realist review of literature. *Disability and Rehabilitation: An International, Multidisciplinary Journal* 2023;45(16):2567-77.

Davey CJ, Landy MSH, Pecora A, et al. A realist review of brief interventions for alcohol misuse delivered in emergency departments. *Systematic Reviews* 2015;4(1):45.

De Salis HF, Martin R, Mansoor Z, et al. A realist review of residential treatment for adults with substance use disorder. *Drug and Alcohol Review* 2023;42(4):827-42.

Garn SD, Glumer C, Villadsen SF, et al. Understanding the mechanisms generating outcomes in a Danish peer support intervention for socially vulnerable people with type 2-diabetes: a realist evaluation. *Archives of Public Health* 2021;79(1):160.

Han S, Zhang Y, Yang X, et al. The effectiveness and sustainability of peer support interventions for persons living with HIV: a realist synthesis. *BMJ Global Health* 2023;8(2):02.

Kors JM, Paternotte E, Martin L, et al. Factors influencing autonomy supportive consultation: A realist review. *Patient Education and Counseling* 2020;103(10):2069-77.

Marshall P, Booth M, Coole M, et al. Understanding the Impacts of Online Mental Health Peer Support Forums: Realist Synthesis. *JMIR Mental Health* 2024;11:e55750.

Maula A, Kendrick D, Kai J, et al. Evidence generated from a realist synthesis of trials on educational weight loss interventions in type 2 diabetes mellitus. *Diabetic Medicine* 2021;38(1):e14394.

Pardoel ZE, Reijneveld SA, Lensink R, et al. Core health-components, contextual factors and program elements of community-based interventions in Southeast Asia - a realist synthesis regarding hypertension and diabetes. *BMC Public Health* 2021;21(1):1917.

Schnabel S, van Wijck F, Kidd L. Supporting stroke survivors to meet their personal rehabilitation needs in community-based arm rehabilitation: development of initial programme theories to explore what may work for whom, how and under what circumstances. *Frontiers in Neurology* 2023;14:1089547.

Stenov V, Lindgreen P, Willaing I, et al. Testing an analogue game to promote peer support and person-centredness in education for people with diabetes: A realist evaluation. *Nursing Open* 2021;8(5):2536-50.

Trickey H, Thomson G, Grant A, et al. A realist review of one-to-one breastfeeding peer support experiments conducted in developed country settings. *Maternal and Child Nutrition* 2017;14(1):e12559.
