# Supplementary material for: Group‐based interventions to reduce diabetes distress in adults with type 1 diabetes: A rapid realist review of the Reduce and TunedIn interventions
Source: Diabet Med. 2026 Feb 12;43(9):e70242. doi: 10.1111/dme.70242 (PMC13614002; doi:10.1111/dme.70242)
Supplement: Supplementary file 2 [file DME-43-e70242-s002.docx]

**Supplementary material 3 - A visual example of how initial programme theories might map onto their contextual factors and outcomes**

| **Initial programme theory** | **C** | **M** | **O** |
| --- | --- | --- | --- |
| **Emotional regulation** | Limited opportunity or ability to talk | Taught how to identify and process unspoken feelings | Greater emotional clarity |
| **Peer learning and support** | Limited peer interaction prior to Reduce | Validation and connection fostered | Reduced feelings of isolation |
| **Person-centred facilitation** | Collaborative rather than authoritative  facilitators | Trust and shared responsibility fostered | Feeling respected and empowered |
| **Diabetes self-management education** | Participant already has high emotional  regulation skills | New knowledge can be absorbed and applied | Improved ability to manage diabetes |
| **Regular assessment and follow-up** | Diabetes distress regularly assessed and  followed up | Emotional wellbeing legitimised as  integral component of diabetes management | Improved clarity and management of diabetes |
| **Motivation for action** | Individual motivation levels responded to by facilitators | Self-direction promoted through personalised support | Increased internal motivation, greater self-regulation |
| **Empowerment and self-management** | Adults with type 1 diabetes positioned as  primary decision-makers in own care | Mindset shift and lived experience recognised | Sense of control, self-efficacy and positive outlook towards diabetes management |
