# Supplementary material for: Group‐based interventions to reduce diabetes distress in adults with type 1 diabetes: A rapid realist review of the Reduce and TunedIn interventions
Source: Diabet Med. 2026 Feb 12;43(9):e70242. doi: 10.1111/dme.70242 (PMC13614002; doi:10.1111/dme.70242)
Supplement: Supplementary file 3 [file DME-43-e70242-s003.docx]

**Supplementary material 4: List of D-Stress Collaborators (not for publication)**

- Iliatha Papachristou Nadal,
- Jennifer Mohammadi,
- Jorg Huber,
- Gary Hickey,
- Pratik Choudhary,
- Sufyan Hussain,
- Kate Hardenberg,
- Francesca Fiorentino,
- Samuel Watson,
- Efrat Lusky,
- Rosie Walker,
- Ramzi Ajjan,
- James Shearer
- Marietta Stadler
