# Supplementary material for: Group‐based interventions to reduce diabetes distress in adults with type 1 diabetes: A rapid realist review of the Reduce and TunedIn interventions
Source: Diabet Med. 2026 Feb 12;43(9):e70242. doi: 10.1111/dme.70242 (PMC13614002; doi:10.1111/dme.70242)
Supplement: Supplementary file 4 [file DME-43-e70242-s004.docx]

**Supplementary material 2.** **Twenty-seven papers included in the rapid realist review**

|  | **Author(s)** | **Date** | **Title** | **Type of document** | **Country of publication** | **Identified at Search Stage:** | **Primarily relevant to** |
| --- | --- | --- | --- | --- | --- | --- | --- |
| 1. | Bendig et al | 2021 | ACTonDiabetes – a guided psychological internet intervention based on Acceptance and Commitment Therapy (ACT) for adults living with Type 1 or 2 diabetes: results of a randomised controlled feasibility trial | Research article | Germany | 2 | RCTs of diabetes distress-related interventions |
| 2. | Due-Christensen et al | 2012 | Can sharing experiences in groups reduce the burden of living with diabetes, regardless of glycaemic control? | Research article | Denmark | 1 | Danish REDUCE |
| 3. | Evans et al | 2024 | Diverse elements comprising studies of peer support complicate evidence synthesis | Research article | USA | 3 | Realist reviews of psychological theories |
| 4. | Fisher et al | 2014 | The confusing tale of depression and distress in patients with diabetes: a call for greater clarity and precision | Review | USA | 1 | TUNED-IN |
| 5. | Fisher et al | 2018a | T1-REDEEM: A Randomized Controlled Trial to Reduce Diabetes Distress Among Adults With Type 1 Diabetes. | Research article | USA | 1 | TUNED-IN |
| 6. | Fisher et al | 2018b | Emotion regulation contributes to the development of diabetes distress among adults with type diabetes | Research article | USA | 1 | TUNED-IN |
| 7. | Fisher et al | 2017 | A practical framework for encouraging and supporting positive behaviour change in diabetes | Review | USA | 1 | TUNED-IN |
| 8. | Fisher et al | 2019a | Addressing diabetes distress in clinical care: a practical guide | Review | USA | 1 | TUNED-IN |
| 9. | Fisher et al | 2019b | Toward effective interventions to reduce diabetes distress among adults with type 1 diabetes: Enhancing Emotion regulation and cognitive skills | Research article | USA | 1 | TUNED-IN |
| 10. | Fisher | 2024a | Clinical Strategies For Addressing The Emotional Side Of Diabetes | PowerPoint presentation | USA | 1 | TUNED-IN |
| 11. | Fisher et al | 2024b | Bringing the Assessment and Treatment of Diabetes Distress into the Real World of Clinical Care: Time for a Shift in Perspective (embargoed) | Research article | USA | 1 | TUNED-IN |
| 12. | Funnell and Anderson | 2004 | Empowerment and self-management of diabetes. | Discussion paper | USA | 1 | Danish REDUCE |
| 13. | Hendrieckx et al | 2020 | Adults With Diabetes Distress Often Want to Talk With Their Health Professionals About It: Findings From an Audit of 4 Australian Specialist Diabetes Clinics | Research article | Australia | 1 | Danish REDUCE |
| 14. | Hessler et al | 2021 | Reductions in management distress following a randomized distress intervention are associated with improved diabetes behavioral and glycemic outcomes over time | Research article | USA | 1 | TUNED-IN |
| 15. | Hessler et al | 2024 | EMBARK—A Randomized, Controlled Trial Comparing Three Approaches to Reducing Diabetes Distress and Improving HbA1c in Adults with Type 1 Diabetes | Research article | USA | 1 | TUNED-IN |
| 16. | Joensen et al | 2017 | The effect of peer support in adults with insulin pump-treated type 1 diabetes: a pilot study. | Research article | Denmark | 1 | Danish REDUCE |
| 17. | McLeish et al | 2023 | Community-based perinatal mental health peer support: a realist review | Realist review | UK | 3 | Realist reviews of psychological theories |
| 18. | McLeish et al | 2024 | A realist change model for community-based perinatal mental health peer support from peer volunteers | Realist review | UK | 3 | Realist reviews of psychological theories |
| 19. | Ryan and Deci | 2000 | Self-determination theory and the facilitation of intrinsic motivation, social development and well-being. | Discussion paper | USA | 1 | Danish REDUCE |
| 20. | Stenov et al | 2024a | A nurse-led intervention to detect and reduce diabetes distress among adults with type 1 diabetes at public funded hospitals in Denmark | PowerPoint presentation | Denmark | 1 | Danish REDUCE |
| 21. | Stenov et al | 2023 | Significant reduction in diabetes distress and improvements in psychosocial outcomes: A pilot test of an intervention to reduce diabetes distress in adults with type 1 diabetes and moderate-to-severe diabetes distress (REDUCE) | Research article | Denmark | 1 | Danish REDUCE |
| 22. | Stenov et al | 2024b | An evidence-based nurse-led intervention to reduce diabetes distress among adults with type 1 diabetes and diabetes distress (REDUCE): Development of a complex intervention informed by the Medical Research Council framework | Research article | Denmark | 1 | Danish REDUCE |
| 23. | TUNED-IN course materials (1) | No date | Self-compassion | PowerPoint presentation training materials | USA | 1 | TUNED-IN |
| 24. | TUNED-IN course materials (2) | No date | Stepping outside | PowerPoint Presentation training materials | USA | 1 | TUNED-IN |
| 25. | TUNED-IN course materials (3) | No date | Value-based goals TUNED-In | PowerPoint Presentation  training materials | USA | 1 | TUNED-IN |
| 26. | TUNED-IN course materials (4) | No date | Clinical strategies for addressing the emotional side of diabetes | PowerPoint Presentation  training materials | USA | 1 | TUNED-IN |
| 27. | Van Den Bulck et al | 2024 | Professionals’ perspectives on interventions to reduce problematic alcohol use in older adults: a realist evaluation of working elements | Research article | The Netherlands | 3 | Realist reviews of psychological theories |
